# Association between serum estradiol level, sex hormone binding globulin level, and bone mineral density in middle-aged postmenopausal women

**DOI:** 10.1186/s13018-021-02799-3

**Published:** 2021-10-30

**Authors:** Zhongxin Zhu, Jin Zhao, Yanfei Fang, Rongwei Hua

**Affiliations:** 1 Department of Clinical Research, The First People’s Hospital of Xiaoshan District, Xiaoshan Affiliated Hospital of Wenzhou Medical University, Hangzhou, 311200 Zhejiang China; 2 Department of Osteoporosis Care and Control, The First People’s Hospital of Xiaoshan District, Xiaoshan Affiliated Hospital of Wenzhou Medical University, Hangzhou, 311200 Zhejiang China; 3 Department of Gynaecology, The First People’s Hospital of Xiaoshan District, Xiaoshan Affiliated Hospital of Wenzhou Medical University, Hangzhou, 311200 Zhejiang China; 4 School of Medical Imaging, Hangzhou Medical College, Hangzhou, 310053 Zhejiang China

**Keywords:** Sex hormones, Estrogen, Sex hormone binding globulin, Postmenopausal women, Bone health

## Abstract

**Background:**

Changes in sex hormones are thought to play an important role in bone health in postmenopausal women. Our aim in this study was to evaluate the association between levels of estradiol (E2), which is the most potent endogenous estrogen, and sex hormone binding globulin (SHBG) and bone mineral density (BMD) among postmenopausal women, 40–59 years of age.

**Methods:**

Using data from the National Health and Nutrition Examination Survey 2013–2016, we performed weighted multivariable linear regression models to evaluate the associations between serum levels of E2 and SHBG and lumbar BMD. A weighted generalized additive model and smooth curve fitting were used to address potential nonlinearity.

**Results:**

A total of 608 postmenopausal women were included in the analysis. The serum E2 level was positively associated with lumbar BMD, after adjusting for other covariates (*β* 0.65; 95% confidence interval (CI) 0.38–0.93). An inverted U-shaped association between the serum E2 level and lumbar BMD was further identified, with the point of inflection at an E2 level of 70 pg/mL. There was no significant association between the SHBG level and lumbar BMD (*β* 0.01; 95% CI − 0.30 to 0.31). However, the association between these two variables was U-shaped, with the point of inflection at an SHBG level of 65 nmol/L.

**Conclusions:**

Based on our findings, it may be beneficial to appropriately increase serum E2 levels to promote bone health in postmenopausal women with low estrogen levels. Considering the inverted U-shaped association, an excessive E2 level may be harmful to BMD. In addition, increasing the SHBG level to within the normal range (65–144 nmol/L) may be considered.

## Background

Osteoporosis is a common systemic musculoskeletal disorder associated with aging, which results in increased disability, mortality, and health-care costs; as such, osteoporosis is a serious public health issue worldwide [1–3]. Postmenopausal osteoporosis, which is the most common type of primary osteoporosis, is mainly caused by an aging-related estrogen deficiency and is associated with a high socioeconomic burden [4, 5]. Bone is highly dynamic, with continuous processes of ossification and resorption to maintain tissue homeostasis [6]. Changes in sex hormones play an important role in bone health among postmenopausal women, with estrogen deficiency after menopause negatively impacting bone remodeling via skeletal and extraskeletal mechanisms [7, 8]. Estrogen deficiency stimulates osteoclast activity by increasing the release of bone-resorbing cytokines, with a rapid bone loss in the early years after menopause and the rate slowing with advancing age [9]. Hormone replacement therapy is one of the treatments used to prevent osteoporosis in postmenopausal, generally being recommended for postmenopausal women under the age of 60 years [10].

Sex hormone-binding globulin (SHBG), which is produced and secreted by the liver, binds sex steroids to regulate their bioavailability in the bloodstream and is another important sex hormone involved in age-related bone health [11, 12]. To date, however, studies have evaluated the association between SHBG and bone mineral density (BMD) among males, with an inverse association between these two variables having been identified [13–15]. Our aim in this study was to evaluate the association between BMD and serum levels of estradiol (E2), which is the most potent endogenous estrogen, and SHBG, among postmenopausal women 40–59 years of age, using a population-based database.

## Materials and methods

### Data source and study population

The National Health and Nutrition Examination Survey (NHANES) is a large, ongoing cross-sectional survey designed to provide objective data on health conditions and address emerging public health issues in the general population in the United States. The survey protocols were approved by the Institutional Review Board of the National Center for Health Statistics, and all participants entered in the NHANES provided consent for the data to be used for research.

Data from the NHANES between 2013 and 2016 were pooled for this study. The study population was restricted to postmenopausal women, 40–59 years of age. Among the 2040 eligible women, we excluded 840 who reported having a regular period in the past 12 months, 287 with an unrecorded menopausal status, 69 with missing serum E2 level data, 66 with missing SHBG level data, 105 with missing lumbar BMD data, and 65 who had a cancer diagnosis. Ultimately, 608 participants were included in the analysis.

### Study variables

Serum E2 levels were measured using isotope dilution liquid chromatography tandem mass spectrometry, based on the reference method of the National Institute for Standards and Technology. SHBG levels were quantified by immuno-antibodies and chemiluminescence measurements. Lumbar BMD was quantified using dual-energy X-ray absorptiometry scans acquired on the Hologic Discovery model A densitometers. Multivariate models contain covariates that might confound the associations between serum E2, SHBG levels and lumbar BMD***.*** The covariates included in this study were age, race, educational level, body mass index, ratio of family income to poverty, moderate activities, smoking at least 100 cigarettes over the life period to the point of data capture, having ≥ 12 alcohol drinks per year over the life period to the point of data capture, blood urea nitrogen, serum uric acid, serum phosphorus, and serum calcium. The detailed process of these variables can be found on the NHANES website (https://www.cdc.gov/nchs/nhanes/).

### Statistical analyses

The study participants were stratified into quartiles according to serum E2 or SHBG levels. All analyses were performed using R software (version 3.4.3), and EmpowerStats software (http://www.empowerstats.com), with statistical significance set at *P* < 0.05. Weighted multivariable linear regression models were used to evaluate the association between serum E2 and SHBG levels and lumbar BMD. According to the Strengthening the Reporting of Observational Studies in Epidemiology (STROBE) statement [16], we conducted three models: Model 1, no adjustment for covariates; Model 2, adjusted for age and race; and Model 3, adjusted for all covariates. A weighted generalized additive model and smooth curve fitting were used to address the potential nonlinearity. Two-piecewise linear regression models were applied to examine threshold effects when nonlinearity associations were found.

## Results

Baseline characteristics of the 608 postmenopausal women included in our study sample, classified by quartiles of serum E2 and SHBG levels, are presented in Tables 1 and 2, respectively. As shown in Table 1, compared to the Q4 group, women with lower serum E2 levels were older and had a lower lumbar BMD. In Table 2, the distribution of age was similar in the different SHBG level groups (*P* > 0.05). Women in the Q3 group of SHBG level had the lowest lumbar BMD.

**Table 1:** Weighted characteristics of study population based on serum estradiol level quartiles

Serum estradiol level (pg/mL)	Q1 (≤ 4.04)	Q2 (4.08–7.58)	Q3 (7.59–16.10)	Q4 (≥ 16.30)	*P* value
Age (years)	54.0 ± 4.3	53.8 ± 4.1	53.2 ± 4.4	50.2 ± 5.0	< 0.001
*Race/Ethnicity (%)*					0.631
Non-Hispanic White	61.4	69.9	68.9	70.4	
Non-Hispanic Black	11.3	12.8	12.1	12.1	
Mexican American	10.1	8.3	7.5	5.8	
Other race/ethnicity	17.1	9.0	11.5	11.7	
*Education level (%)*					0.018
Less than high school	18.3	16.0	12.2	10.0	
High school	27.3	16.1	29.3	20.9	
More than high school	54.4	67.9	58.5	69.1	
Body mass index (kg/m^2^)	26.4 ± 5.6	29.9 ± 5.8	33.4 ± 6.1	32.2 ± 8.5	< 0.001
Income to poverty ratio	2.9 ± 1.8	3.2 ± 1.7	3.2 ± 1.5	3.4 ± 1.6	0.096
*Moderate activities (%)*					0.942
Yes	42.7	45.3	46.3	44.8	
No	57.3	54.7	53.7	55.2	
*Smoked at least 100 cigarettes in life (%)*					0.018
Yes	40.3	48.9	46.4	33.1	
No	59.7	51.1	53.6	66.9	
*Had at least 12 alcohol drinks in a year (%)*					0.174
Yes	68.2	75.0	65.6	72.5	
No	31.8	25.0	34.4	27.5	
Blood urea nitrogen (mg/dL)	5.0 ± 1.6	5.1 ± 1.8	4.9 ± 1.8	4.6 ± 1.2	0.036
Serum uric acid (umol/L)	269.4 ± 68.0	297.3 ± 72.0	301.1 ± 72.5	291.6 ± 60.9	< 0.001
Serum phosphorus (mg/dL)	1.3 ± 0.2	1.3 ± 0.1	1.2 ± 0.2	1.2 ± 0.2	< 0.001
Serum calcium (mg/dL)	2.4 ± 0.1	2.4 ± 0.1	2.4 ± 0.1	2.3 ± 0.1	0.005
Sex hormone binding globulin (nmol/L)	75.6 ± 36.6	61.7 ± 45.7	49.6 ± 31.6	70.6 ± 43.2	< 0.001
Lumbar bone mineral density (mg/cm^2^)	936.2 ± 138.8	964.3 ± 137.3	994.4 ± 136.6	1051.7 ± 141.0	< 0.001

Mean ± SD for continuous variables: *P* value was calculated by weighted linear regression model. % for Categorical variables: *P* value was calculated by weighted chi-square test

**Table 2:** Weighted characteristics of study population based on sex hormone binding globulin quartiles

Sex hormone binding globulin level (nmol/L)	Q1 (≤ 35.96)	Q2 (35.97–52.0)	Q3 (52.08–75.52)	Q4 (≥ 76.23)	*P* value
Age (years)	51.9 ± 5.0	53.0 ± 5.0	52.8 ± 4.6	52.9 ± 4.5	0.200
*Race/Ethnicity (%)*					0.008
Non-Hispanic White	67.8	58.4	62.6	79.5	
Non-Hispanic Black	10.0	16.4	13.5	9.4	
Mexican American	8.9	11.8	8.0	3.7	
Other race/ethnicity	13.3	13.5	15.9	7.3	
*Education level (%)*					0.006
Less than high school	10.5	16.4	18.2	11.0	
High school	30.4	27.9	20.9	16.1	
More than high school	59.1	55.7	60.9	72.9	
Body mass index (kg/m^2^)	34.6 ± 7.0	31.5 ± 6.4	30.4 ± 7.3	27.2 ± 6.0	< 0.001
Income to poverty ratio	3.0 ± 1.5	3.1 ± 1.6	3.3 ± 1.8	3.2 ± 1.7	0.309
*Moderate activities (%)*					0.099
Yes	39.9	45.6	40.4	51.9	
No	60.1	54.4	59.6	48.1	
*Smoked at least 100 cigarettes in life (%)*					0.116
Yes	41.6	42.8	34.5	47.6	
No	58.4	57.2	65.5	52.4	
*Had at least 12 alcohol drinks in a year (%)*					< 0.001
Yes	57.5	75.9	62.5	83.2	
No	42.5	24.1	37.5	16.8	
Blood urea nitrogen (mg/dL)	5.1 ± 1.6	4.9 ± 1.5	4.7 ± 1.6	4.9 ± 1.7	0.107
Serum uric acid (umol/L)	324.0 ± 64.1	301.5 ± 68.3	285.1 ± 62.8	260.8 ± 65.2	< 0.001
Serum phosphorus (mg/dL)	1.2 ± 0.2	1.2 ± 0.2	1.2 ± 0.2	1.3 ± 0.1	0.035
Serum calcium (mg/dL)	2.4 ± 0.1	2.4 ± 0.1	2.4 ± 0.1	2.4 ± 0.1	0.517
Serum estradiol level (pg/mL)	23.6 ± 37.7	17.8 ± 37.2	27.1 ± 45.9	28.6 ± 48.2	0.140
Lumbar bone mineral density (mg/cm^2^)	1011.6 ± 137.4	998.2 ± 156.4	962.9 ± 140.6	991.4 ± 142.6	0.032

Mean ± SD for continuous variables: *P* value was calculated by weighted linear regression model. % for Categorical variables: *P* value was calculated by weighted chi-square test

The association between serum E2 level and lumbar BMD was positive in all three regression models (Table 3): model 1 (*β* 0.80; 95% confidence interval (CI) 0.54–1.06); model 2 (*β* 0.71; 95% CI 0.44–0.98); model 3 (*β* 0.65; 95% CI 0.38–0.93). The *P* value was significant for all three models (*P* < 0.001). There was no significant association between the SHBG level and lumbar BMD, as follows (Table 4): model 1 (*β* − 0.05; 95% CI − 0.34 to 0.23); model 2 (*β* − 0.13; 95% CI − 0.41 to 0.14); model 3 (*β* 0.01; 95% CI − 0.30 to 0.31). The *P* values for these regressions were not significant.

**Table 3:** Association between serum estradiol level (pg/mL) and lumbar bone mineral density (mg/cm^2^)

	Model 1 *β* (95% CI)	Model 2 *β* (95% CI)	Model 3 *β* (95% CI)
Serum estradiol level	0.80 (0.54, 1.06)***	0.71 (0.44, 0.98)***	0.65 (0.38, 0.93)***
*Serum estradiol level categories*			
Q1	Reference	Reference	Reference
Q2	28.09 (− 4.47, 60.66)	22.62 (− 9.46, 54.70)	9.48 (− 23.99, 42.96)
Q3	58.25 (25.96, 90.53)	53.09 (21.27, 84.92)	36.44 (1.96, 70.93)
Q4	115.57 (84.12, 147.02)	107.49 (75.11, 139.86)	90.98 (55.80, 126.16)
*P* for trend	< 0.001	< 0.001	< 0.001

Model 1: no covariates were adjusted. Model 2: age, and race were adjusted. Model 3: age, race, educational level, body mass index, ratio of family income to poverty, moderate activities, smoked at least 100 cigarettes in life, had at least 12 alcohol drinks in a year, blood urea nitrogen, serum uric acid, serum phosphorus, and serum calcium were adjusted

*P < 0.05; **P < 0.01; ***P < 0.001

**Table 4:** Association between sex hormone binding globulin level (nmol/L) and lumbar bone mineral density (mg/cm^2^)

	Model 1 *β* (95% CI)	Model 2 *β* (95% CI)	Model 3 *β* (95% CI)
Sex hormone binding globulin	− 0.05 (− 0.34, 0.23)	− 0.13 (− 0.41, 0.14)	0.01 (− 0.30, 0.31)
*Sex hormone binding globulin categories*			
Q1	Reference	Reference	Reference
Q2	− 13.36 (− 47.43, 20.71)	− 9.25 (− 42.62, 24.12)	− 15.19 (− 49.77, 19.40)
Q3	− 48.66 (− 81.85, − 15.48)	− 47.00 (− 79.39, − 14.62)	− 42.37 (− 76.34, − 8.41)
Q4	− 20.17 (− 51.88, 11.54)	− 24.51 (− 55.61, 6.60)	− 13.86 (− 49.23, 21.51)
*P* for trend	0.094	0.039	0.260

Model 1: no covariates were adjusted. Model 2: age, and race were adjusted. Model 3: age, race, educational level, body mass index, ratio of family income to poverty, moderate activities, smoked at least 100 cigarettes in life, had at least 12 alcohol drinks in a year, blood urea nitrogen, serum uric acid, serum phosphorus, and serum calcium were adjusted

*P < 0.05; **P < 0.01; ***P < 0.001

**Fig. 1:**
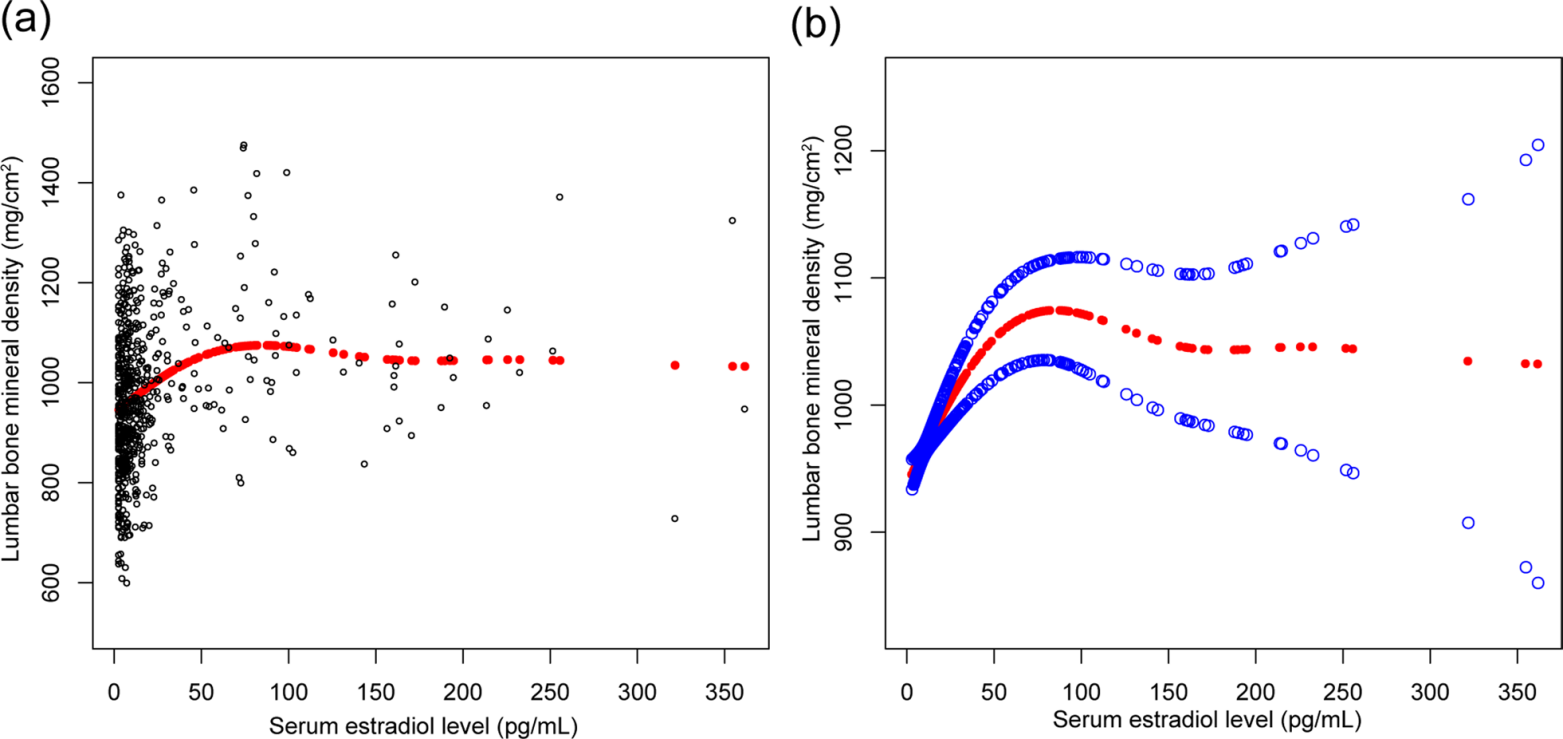
The associations between serum estradiol level and lumbar bone mineral density. **a** Each black point represents a sample. **b** Solid red line represents the smooth curve fit between variables. Blue bands represent the 95% of confidence interval from the fit. Adjusted for age, race, educational level, body mass index, ratio of family income to poverty, moderate activities, smoked at least 100 cigarettes in life, had at least 12 alcohol drinks in a year, blood urea nitrogen, serum uric acid, serum phosphorus, and serum calcium

**Fig. 2:**
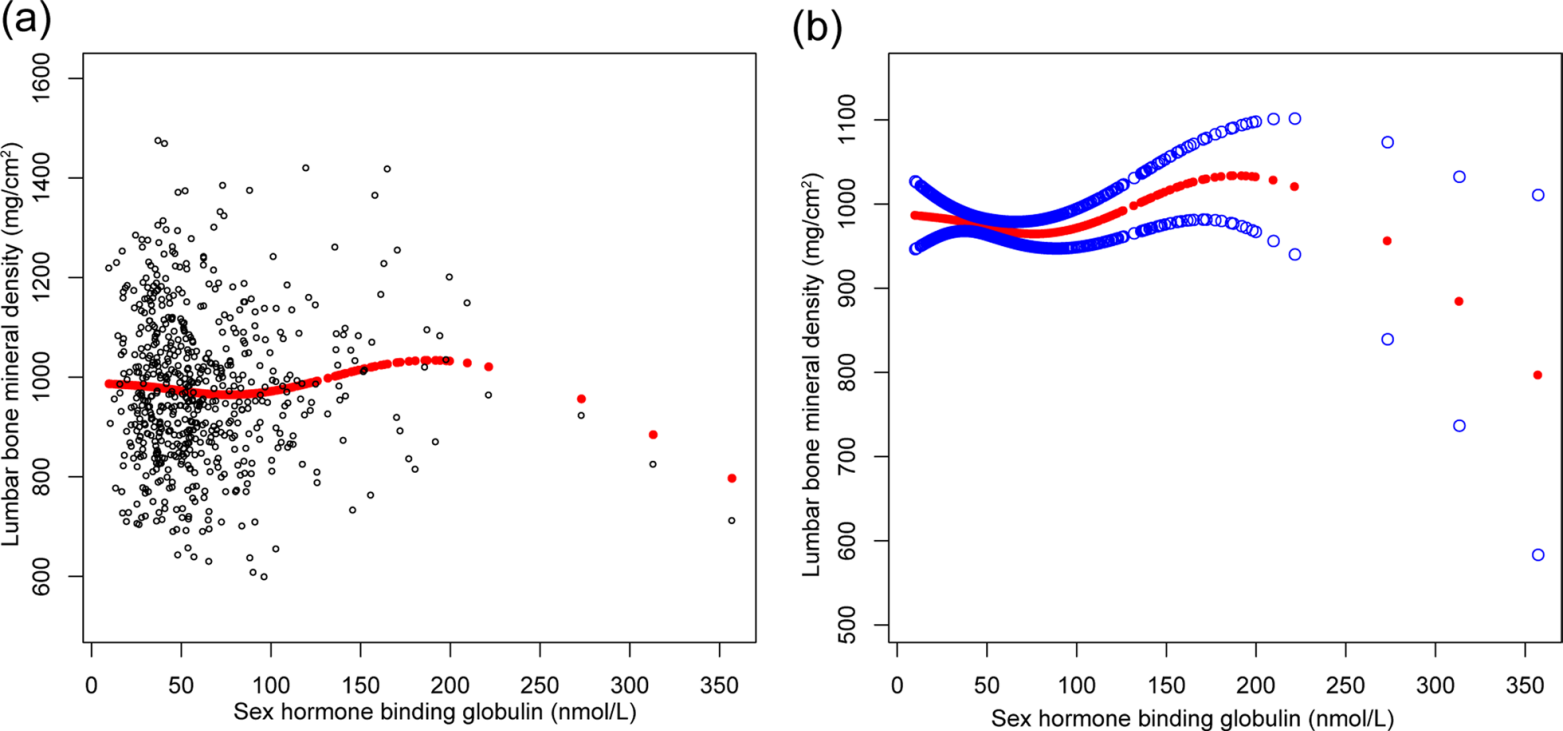
The associations between SHBG level and lumbar bone mineral density. **a** Each black point represents a sample. **b** Solid red line represents the smooth curve fit between variables. Blue bands represent the 95% of confidence interval from the fit. Adjusted for age, race, educational level, body mass index, ratio of family income to poverty, moderate activities, smoked at least 100 cigarettes in life, had at least 12 alcohol drinks in a year, blood urea nitrogen, serum uric acid, serum phosphorus, and serum calcium

The nonlinear relationship between serum levels of E2 and SHBG and lumbar BMD is shown in Figs. 1 and 2, respectively. Using a two-piecewise linear regression model, the point of inflection in the inverted U-shaped or U-shaped association between measured serum levels and lumbar BMD was at a level of 70 pg/mL for E2 and 65 nmol/L for SHBG (Table 5).

**Table 5:** Threshold effect analysis of serum estradiol level and sex hormone binding globulin level on lumbar bone mineral density using two-piecewise linear regression model

Lumbar bone mineral density	Adjusted *β* (95% CI), *P* value
*Serum estradiol level*	
Fitting by standard linear model	0.65 (0.38, 0.93) < 0.001
*Fitting by two-piecewise linear model*	
Inflection point	70 (pg/mL)
Serum estradiol level < 70 (pg/mL)	1.92 (1.25, 2.59)
Serum estradiol level > 70 (pg/mL)	− 0.24 (− 0.75, 0.27)
Log likelihood ratio	< 0.001
*Sex hormone binding globulin*	
Fitting by standard linear model	0.01 (− 0.30, 0.31) 0.968
*Fitting by two-piecewise linear model*	
Inflection point	65 (nmol/L)
Sex hormone binding globulin < 65 (nmol/L)	− 0.95 (− 1.80, − 0.09) 0.030
Sex hormone binding globulin > 65 (nmol/L)	0.33 (− 0.08, 0.73) 0.115
Log likelihood ratio	0.016

Age, race, educational level, body mass index, ratio of family income to poverty, moderate activities, smoked at least 100 cigarettes in life, had at least 12 alcohol drinks in a year, blood urea nitrogen, serum uric acid, serum phosphorus, and serum calcium were adjusted

## Discussion

In our study sample, which was a national representation of middle-aged postmenopausal women, the serum E2 level was positively associated with lumbar BMD, with no significant association between the SHBG level and lumbar BMD. Of note, we identified an inverted U-shaped association between BMD and serum E2, with a U-shaped association between BMD and serum SHBG.

A decline in E2 level has been recognized as the most critical hormonal regulator of the menopause-associated decrease in BMD [17]. A study from Spain reported a positive association between E2 levels and BMD [18]. In a study of 132 women with postmenopausal osteoporosis and 81 postmenopausal women without osteoporosis, serum concentrations of E2 were found to be significantly lower in the osteoporosis group, indicative of a positive correlation between E2 and BMD [7]. A recent genome-wide study provided further support of the effects of E2 on BMD in maintaining skeletal health in both men and women [19]. In contrast, we identified an inverted U-shaped association between BMD and serum E2 levels, with a point of inflection at 70 pg/mL. The inverted U-shape indicates that an excessive E2 level may be harmful to BMD. Further prospective intervention trials are warranted to confirm this conclusion.

A previous study identified that a higher SHBG level may be a risk factor for osteoporosis [20]. Evidence from the Concord Health and Ageing in Men Project in Australia reported that increasing serum SHBG levels were significantly associated with lower hip BMD [13]. A cross-sectional study of 404 men ≥ 45 years of age in China reported an inverse relationship between SHBG levels and BMD [14]. An inverse relationship between the SHBG level and BMD was also reported in the Osteoporotic Fractures in Men study, which included 1500 community-dwelling older men [15]. As well, a cross-sectional study reported a negative association between serum SHBG levels and bone mass, measured using quantitative ultrasound, among 382 premenopausal women [21]. This body evidence indicates that SHBG might play an important role in the development of osteoporosis, although this association may be influenced by skeletal site and age [22]. In contrast, as for the relationship between E2 and lumbar BMD, we identified a U-shaped association between SHBG and lumbar BMD, with the point of inflection at 65 nmol/L. A previous study reported that a lower SHBG level is associated with several diseases, including liver disease, arthritis, polycystic ovarian syndrome, cancer, and cardiovascular disease [23]. Therefore, properly increasing SHBG levels within the normal range (65–144 nmol/L) may be considered.

The NHANES data are collected following standardized protocols, which could assure the accuracy and consistency of our data and results. However, the limitations of our study should be acknowledged in the interpretation of our results. First, a causal inference cannot be derived due to the cross-sectional design of the NHANES survey. A large-scale cohort study is necessary to further strengthen our results. Second, the NHANES samples were only measured once, which could have led to potential bias. Therefore, multiple tests are recommended for future studies. Third, although we used a nationally representative sample in this study, the population was restricted to postmenopausal women, 40–59 years of age. Therefore, the conclusions in this study cannot be generalized to premenopausal women or edlerly women.

## Conclusion

Our finding revealed an inverted U-shaped association between serum E2 levels and lumbar BMD, suggesting that it may be beneficial to appropriately increase serum E2 levels to promote bone health in postmenopausal women with low estrogen levels, and an excessive E2 level may be harmful to BMD. In addition, increasing the SHBG level to within the normal range (65–144 nmol/L) may be considered.

## References

[1] Migliorini F, Maffulli N, Colarossi G, Eschweiler J, Tingart M, Betsch M (2021). Effect of drugs on bone mineral density in postmenopausal osteoporosis: a Bayesian network meta-analysis. J Orthop Surg Res.

[2] Migliorini F, Maffulli N, Spiezia F, Peretti GM, Tingart M, Giorgino R (2021). Potential of biomarkers during pharmacological therapy setting for postmenopausal osteoporosis: a systematic review. J Orthop Surg Res.

[3] Conti V, Russomanno G, Corbi G, Toro G, Simeon V, Filippelli W, Ferrara N, Grimaldi M, D'Argenio V, Maffulli N (2015). A polymorphism at the translation start site of the vitamin D receptor gene is associated with the response to anti-osteoporotic therapy in postmenopausal women from southern Italy. Int J Mol Sci.

[4] Ren ZQ, Wang YF, Ao GF, Chen HX, Huang M, Lai MX, Zhao HD, Zhao R (2020). Overall adjustment acupuncture for postmenopausal osteoporosis (PMOP): a study protocol for a randomized sham-controlled trial. Trials.

[5] Migliorini F, Colarossi G, Baroncini A, Eschweiler J, Tingart M, Maffulli N (2021). Pharmacological management of postmenopausal osteoporosis: a level i evidence based—expert opinion. Expert Rev Clin Pharmacol.

[6] Migliorini F, Maffulli N, Spiezia F, Tingart M, Maria PG, Riccardo G (2021). Biomarkers as therapy monitoring for postmenopausal osteoporosis: a systematic review. J Orthop Surg Res.

[7] Mederle OA, Balas M, Ioanoviciu SD, Gurban CV, Tudor A, Borza C (2018). Correlations between bone turnover markers, serum magnesium and bone mass density in postmenopausal osteoporosis. Clin Interv Aging.

[8] Park HS, Kim GY, Lo JA, Kim JS, Ahn SY, Ko GJ, Kwon YJ, Kim JE (2021). Urine and Serum Electrolytes and Biochemical Values Associated with Osteoporosis in Premenopausal and Postmenopausal Women: a Longitudinal and Cross-Sectional Study Using Korean Genome and Epidemiology Study (KoGES) Cohort. J Clin Med.

[9] Zhang Y, Hua F, Ding K, Chen H, Xu C, Ding W (2019). Angiogenesis changes in ovariectomized rats with osteoporosis treated with estrogen replacement therapy. Biomed Res Int.

[10] The 2017 hormone therapy position statement of The North American Menopause Society. *Menopause (New York, NY)* 2017, 24(7):728–53. 10.1097/GME.0000000000000921 28650869

[11] Simó R, Sáez-López C, Barbosa-Desongles A, Hernández C, Selva DM (2015). Novel insights in SHBG regulation and clinical implications. Trends Endocrinol Metab.

[12] Hoppé E, Bouvard B, Royer M, Audran M, Legrand E (2010). Sex hormone-binding globulin in osteoporosis. Joint Bone Spine.

[13] Hsu B, Seibel MJ, Cumming RG, Blyth FM, Naganathan V, Bleicher K, Le Couteur DG, Waite LM, Handelsman DJ (2016). Progressive temporal change in serum SHBG, but not in serum testosterone or estradiol, is associated with bone loss and incident fractures in older men: the concord health and ageing in men project. J Bone Miner Res.

[14] Zha XY, Hu Y, Pang XN, Zhu JH, Chang GL, Li L (2014). Sex hormone-binding globulin (SHBG) as an independent determinant of bone mineral density (BMD) among Chinese middle-aged and elderly men. Endocrine.

[15] Woods GN, Huang MH, Cawthon PM, Laughlin GA, Schousboe JT, McDaniels-Davidson C, Cauley JA, Orwoll E, Barrett-Connor E, Kado DM (2016). SHBG, sex steroids, and kyphosis in older men: the MrOS study. J Bone Miner Res.

[16] von Elm E, Altman DG, Egger M, Pocock SJ, Gøtzsche PC, Vandenbroucke JP (2007). The strengthening the reporting of observational studies in epidemiology (STROBE) statement: guidelines for reporting observational studies. Lancet (London, England).

[17] Park YM, Jankowski CM, Swanson CM, Hildreth KL, Kohrt WM, Moreau KL (2021). Bone mineral density in different menopause stages is associated with follicle stimulating hormone levels in healthy women. Int J Environ Res Public Health.

[18] Zolfaroli I, Ortiz E, García-Pérez M, Hidalgo-Mora JJ, Tarín JJ, Cano A (2021). Positive association of high-density lipoprotein cholesterol with lumbar and femoral neck bone mineral density in postmenopausal women. Maturitas.

[19] Schmitz D, Ek WE, Berggren E, Höglund J, Karlsson T, Johansson Å. Genome-wide association study of estradiol levels, and the causal effect of estradiol on bone mineral density. J Clin Endocrinol Metab. 2021. 10.1210/clinem/dgab507 PMC8530739 34255042

[20] Legrand E, Hedde C, Gallois Y, Degasne I, Boux de Casson F, Mathieu E, Baslé MF, Chappard D, Audran M (2001). Osteoporosis in men: a potential role for the sex hormone binding globulin. Bone.

[21] Wei S, Jones G, Thomson R, Otahal P, Dwyer T, Venn A (2010). Menstrual irregularity and bone mass in premenopausal women: cross-sectional associations with testosterone and SHBG. BMC Musculoskelet Disord.

[22] Qu Z, Jiang J, Yang F, Huang J, Zhao J, Yan S (2021). Genetically predicted sex hormone-binding globulin and bone mineral density: a mendelian randomization study. Calcif Tissue Int.

[23] Wang Y. Definition, prevalence, and risk factors of low sex hormone-binding globulin in US adults. J Clin Endocrinol Metab. 2021. 10.1210/clinem/dgab416 PMC8571812 34125885

